# Musculoskeletal pain distribution in 1,000 Danish schoolchildren aged 8–16 years

**DOI:** 10.1186/s12998-020-00330-9

**Published:** 2020-08-04

**Authors:** Signe Fuglkjær, Werner Vach, Jan Hartvigsen, Kristina Boe Dissing, Tina Junge, Lise Hestbæk

**Affiliations:** 1 Department of Sports Science and Clinical Biomechanics, Faculty of Health Sciences, University of Southern Denmark, Campusvej 55, 5230 Odense M, Denmark; 2 Department of Orthopaedics and Traumatoloy, University Hospital Basel, Spitalstr 21, CH-4031 Basel, Switzerland; 3 Institute for Medical Biometry and Statistics, Medical Faculty and Medical Center, University of Freiburg, Freiburg, Germany; 4 Nordic Institute of Chiropractic and Clinical Biomechanics, Campusvej 55, 5230 Odense M, Denmark; 5 Health Sciences Research Centre, University College Lillebaelt, Niels Bohrs allé 1, 5230 Odense M, Denmark

**Keywords:** Paediatrics, Multi region pain, Children, Prevalence, Occurrence, Adolescence, Distribution

## Abstract

**Background:**

Knowledge about the occurrence and distribution of musculoskeletal problems in early life is needed. The objectives were to group children aged 8 to 16 according to their distribution of pain in the spine, lower- and upper extremity, determine the proportion of children in each subgroup, and describe these in relation to sex, age, number- and length of episodes with pain.

**Method:**

Data on musculoskeletal pain from about 1,000 Danish schoolchildren was collected over 3 school years (2011 to 2014) using weekly mobile phone text message responses from parents, indicating whether their child had pain in the spine, lower extremity and/or upper extremity. Result are presented for each school year individually.

**Results:**

When pain was defined as at least 1 week with pain during a school year, Danish schoolchildren could be divided into three almost equally large groups for all three school years: Around 30% reporting no pain, around 40% reporting pain in one region, and around 30% reporting pain in two or three regions. Most commonly children experienced pain from the lower extremities (~ 60%), followed by the spine (~ 30%) and the upper extremities (~ 23%). Twice as many girls reported pain in all three sites compared to boys (10% vs. 5%) with no other statistically significant sex or age differences observed. When pain was defined as at least 3 weeks with pain during a schoolyear, 40% reported pain with similar patterns to those for the more lenient pain definition of 1 week.

**Conclusion:**

Danish schoolchildren often experienced pain at more than one pain site during a schoolyear, and a significantly larger proportion of girls than boys reported pain in all three regions. This could indicate that, at least in some instances, the musculoskeletal system should be regarded as one entity, both for clinical and research purposes.

## Background

Musculoskeletal (MSK) pain can start early in life [1–3] and increases throughout adolescence [2, 4, 5]. Furthermore, knee pain [6, 7], spinal pain [8] and MSK pain in general [2, 9] have been shown to be recurrent conditions, and multi-site pain may exist already in adolescence [10–12]. Holden et al. categorised adolescents into four classes describing their pain experience and demonstrated that multi-site pain was more common than pain in a single region [10].

MSK pain in children may have a negative impact on sports participation [13–15], and physical activity in childhood is important for childhood and later health [16], highlighting the importance of optimizing MSK health. In addition, MSK problems in children have been linked to psychological distress [17, 18], poor relations with peers [5, 19], absence from school [13], puberty [20, 21] and decreased quality of life [7, 10, 22]. To improve our understanding in this important area, and to learn when and how to intervene, we need more basic epidemiological knowledge about the occurrence and distribution of MSK problems in early life.

MSK pain in different regions of the body (spine, upper extremity (UE) and lower extremity (LE)) has been described in detail in children participating in the Childhood Health, Activity and Motor Performance School Study (CHAMPS Study-DK) [8, 23–26]. LE pain and spinal pain were common and recurrent in the children, whereas UE pain was less common and most often short-lasting [23]. Furthermore, it was found that the frequency of spinal pain increased as the children approached adolescence [8], whereas the frequency of LE pain decreased [23]. The question remains, however, to what extent pain in different body regions overlap in individuals and if specific combinations of pain are prevalent in this age group.

In the current study we therefore aimed to describe the distribution of MSK pain over the course of 1 year in children aged 8 to 16 years, specifically whether MSK pain typically is limited to one region or whether pain presents in more than one region.

Specifically, we wanted to: 1) Categorize children into eight subgroups as having ‘no pain’; ‘spinal pain only’; ‘UE pain only’; ‘LE pain only’; ‘Spinal and UE pain’; ‘Spinal and LE pain’; UE and LE pain’; ‘pain in all regions’, as well as the proportion of children in each of the eight subgroups, 2) Determine sex and age, number of weeks with pain as well as mean number of episodes, and mean length of episodes for the eight groups.

## Method

### Setting

This was a prospective school-based cohort study nested within the CHAMPS Study-DK [27]. The CHAMPS Study-DK started in 2008 and data collection continued until June 2015. CHAMPS Study-DK is a dynamic cohort study as children could enter and leave the study at any time during the study period, and is described in detail elsewhere [27].

### Study population

In August 2011, pupils attending grades three to seven in 13 out of 17 public primary schools in the municipality of Svendborg, Denmark, were invited to participate in the study, and data until June 2014 (three schoolyears) was used in this paper. Svendborg has 58,000 inhabitants and is comparable to the rest of Denmark in terms of age, sex and income, but has a slightly higher unemployment rate (5.3% versus 4.5%) [28]. In Svendborg, 84% of the children attend public schools, and therefore all levels of socioeconomic status were represented.

### Data collection

Information on pain was registered by parent-reported weekly mobile phone text message (SMS) responses. Every week, parents received the following SMS question: ‘Has [name of the child] had any pain during the past week in: 1-Neck or back; 2-Shoulder, arm or hand; 3-Hip, leg or foot; or 4-No, [name of the child] did not have any pain.’ It was possible to report pain in more than one region. If parents did not reply, they received reminders twice with an interval of 48 h. The SMS question was sent out every week except for 6 weeks during the summer holidays (July and August) and 1 week during the Christmas holidays.

*Outcome variables*

- No pain the past week (Y/N)
- Spinal pain the past week (Y/N)
- UE pain the past week (Y/N)
- LE pain the past week (Y/N)

### Case definitions

Various terms have been used to describe pain in more than one anatomical region (spine, upper extremity (UE) or lower extremity (LE)). The term ‘widespread pain’ has traditionally been used to describe pain in more than one region, however this term also includes pain outside of the MSK system such as abdominal pain and/or headache. ‘Multisite pain’ is traditionally defined as pain in more than one anatomical site, and this can be within the same region, or in different regions. As none of these terms fit the purpose of the analyses in this article, we decided to use the term *‘*pain in more than one region’ when pain was located in more than one region during a school year. The pain does not have to co-exist but can be present at the same or different time-points during a school year.

### Statistical analyses

The children were followed for up to 3 years, and results are reported by school year. To obtain a satisfactory observation period, children should participate in the study for at least a full school year minus 1 week and comply with 85% or more valid SMS responses. Potential differences in demographics were tested both between participants and non-participants (dropouts or non-consenters) and between compliers and non-compliers (> 15% missing SMS-responses). For non-participants, only sex was available.

Children were categorized into eight groups for each school year as having either ‘No pain’; three groups with pain in one region: ‘Spinal pain only’, ‘UE pain only’ or ‘LE pain only’; three groups with pain in two regions within a school year: ‘Spinal and UE pain’, ‘Spinal and LE pain’ and ‘UE and LE pain’; and finally one group with pain from both spine, upper- and lower extremities: ‘Pain in all regions’. Proportions of children in each of the eight groups were calculated by school year and reported as percentages with 95% confidence intervals (CI). Each of the eight groups was then described in terms of sex expressed by percentages with 95% CI and age with standard deviations (SD). Further for each group, the proportion of weeks with pain during a school year was calculated and expressed by means with 95% CI.

Length of an episode was defined as consecutive weeks with pain reported in the same region. The number of episodes per child was calculated as the sum of episodes for each child during a school year. Both variables are reported as means with 95% confidence intervals for each group. Since it is unknown if one single report of pain is of importance or whether a certain duration is needed to influence the life of the children, all analyses were performed using two different definitions, i.e. *‘At least 1 pain week’* and *“At least 3 pain weeks’* during one schoolyear.

#### Missing SMS responses

If four or fewer consecutive SMS responses were missing, they were imputed with the same value as the previous week’s response, provided that the response was the same for the week *after* the missing response(s). Otherwise, we defined the end of that episode as occurring at the week prior to the missing response. If there were more than four consecutive missing SMS responses, we also defined the end of the episode as occurring at the week prior to the first missing SMS response. A sensitivity analysis was performed to estimate the impact of these decision rules by treating missing SMS responses in two extreme ways to determine the range within which the correct value would lie: First, we coded missing SMS response to be the same as the last SMS response, regardless of the value of the next response, which would potentially inflate episode lengths and diminish the number of episodes. Second, we coded SMS responses as ‘no pain’ for all weeks with missing SMS responses, which would do the opposite.

STATA 15.0 (StataCorp, College Station, Texas, USA) was used for the analyses. Significance level was set at 0.05, and statistically tests were not used but significant differences was found by the use of overlapping CIs.

## Results

### Study sample

**Fig. 1:**
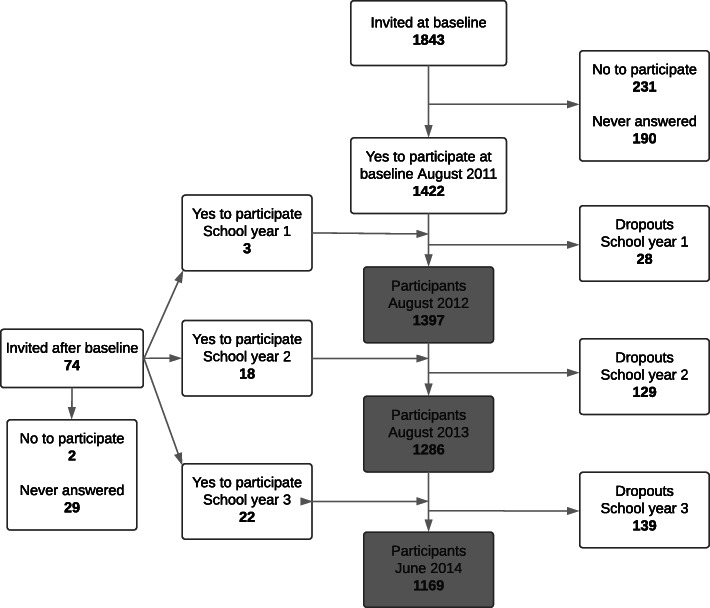
Overview of the participant flow in a cohort of Danish school children (CHAMPS Study-DK; *n* = 1465)

From August 2011 to June 2014, 1917 children were invited to participate in the CHAMPS Study-DK, and 1465 (76%) children were enrolled. During this period 296 children dropped out (Fig. 1).

**Fig. 2:**
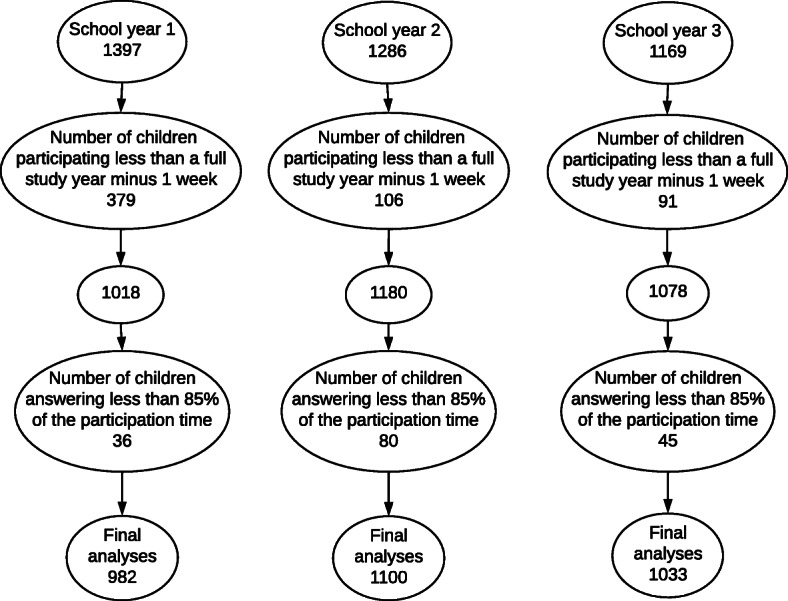
Participant flow after inclusion

The average weekly response rate for all three school years was 96%. After excluding children with low SMS compliance, the final sample consisted of 982 children in school year 1, 1100 children in school year 2 and 1033 children in school year 3 (Fig. 2). The total number of SMS responses was 43,171; 51,641 and 47,495 for the three school years, respectively.

In August 2011, children were 8 to 14 years of age (mean 10.7 (SD 1.4)), and during all three school years, 52% were girls (Table 1).

**Table 1:** Age, sex and pain prevalence by school year in a cohort of Danish school children (CHAMPS Study-DK)

	School year 1 (August 2011 to June 2012)	School year 2 (August 2012 to June 2013)	School year 3 (August 2013 to June 2014)
Total	982 (100.0)	1100 (100.0)	1033 (100.0)
Girls, n (%)	512 (52.1)	576 (52.4)	539 (52.2)
Mean age, years (SD)	10.7 (SD 1.4)	11.6 (SD 1.4)	12.5 (SD 1.4)
Spinal pain, % (95% CI)	28.8 (26.1; 31.7)	33.6 (30.9; 36.5)	31.2 (28.4; 34.1)
Upper extremity pain, % (95% CI)	22.6 (20.1; 25.3)	23.8 (21.4; 26.4)	22.0 (19.6; 24.6)
Lower extremity pain, % (95% CI)	60.1 (57.0; 63.1)	58.3 (55.3; 61.2)	53.1 (50.1; 56.2)
No pain, % (95% CI)	28.5 (25.8; 31.4)	27.1 (24.5; 29.8)	30.8 (28.0; 33.7)

About 30% of the children did not experience any pain during a school year (Table 1). When reporting pain it was most frequently in the LEs, 53.1% (95% CI 50.1; 56.2) to 60.1% (95% CI 57.0; 63.1), whereas they least frequently reported pain in the UEs, 22.0% (95% CI 19.6; 24.6) to 23.8% (95% CI 21.4; 26.4).

There were no significant differences between the children who declined participation, had low SMS compliance or dropped out when compared to the study sample in relation to sex, but the dropouts were on average older than the children who remained in the study (12.5 versus 10.6 years of age at baseline, *p* < 0.001).

### Pain patterns

Findings are presented in detail for school year 1, followed by a description of differences for schoolyear 2 and 3. Tables and figures with detailed information pertaining to school years 2 and 3 can be seen in Additional file 1.

### Results for ‘At least one pain week’ during a school year

#### Distribution of children into eight pain groups

**Fig. 3:**
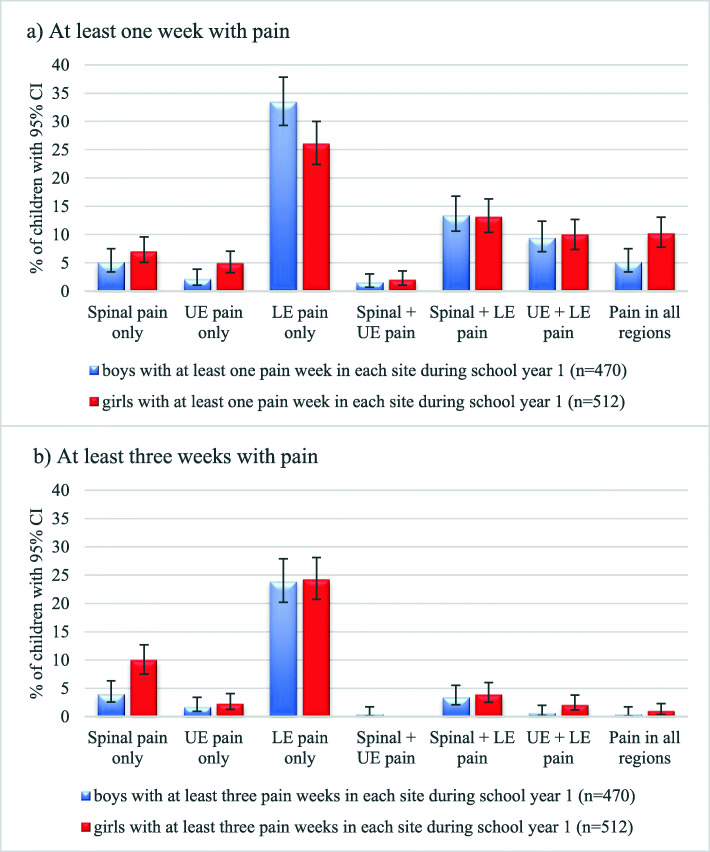
Proportion of Danish children aged 8 to 16 years with either a) at least 1 week with pain or b) at least 3 weeks with pain in each anatomical region during school year 1. Presented by sex and with 95% confidence interval (CI). UE: upper extremity, LE: lower extremity

Using this definition, 28.5% of the children did not experience pain during school year 1. The 71.5% of the children who did experience pain, most commonly did so in the LE (60.0%), followed by spinal pain (28.7%), and the UE (22.6%). For those reporting pain, it was most commonly in one region only (39.2%) and most often in the LE (29.5%). Among those reporting pain, pain in more than one region was, however, also common (32.2%), most commonly spinal and LE pain (13.2%). The distribution of the subgroups is shown in Fig. 3a.

These patterns remained for the other two school years with 72.9 and 69.2% reporting pain during year 2 and 3, respectively, LE pain being most common, and about one third reporting pain at more than one site (Add. file 1).

#### Sex

In school year 1, boys reported more LE pain only than girls, whereas the girls reported more spinal pain and UE pain. Pain at two sites was approximately equally distributed between the two sexes, whereas pain at all three sites was more common in girls (Fig. 3a).

The sex difference for the group with pain at all three sites remains across the 3 years, but other differences in sex distribution become progressively smaller over the three-year period (Add. file 1).

#### Age

Generally, there were no differences in mean age between the sub-groups (Table 2 and Add. file 1).

**Table 2:** Description of Danish children aged 8 to 16 years during school year 1. Reported by pain group. Pain was defined as **at least one pain week** from a body region during a school year

Pain site(s)	Proportion % (95%CI)	Age Years (SD)	Sex % female	Weeks with pain % (95%CI)	Number of episodes (95%CI)	Length of episodes Weeks (95%CI)
No pain (*n* = 279)	28.5 (25.7; 31.3)	10.6 (1.5)	49.6	0	0	-
SP only (*n* = 60)	6.1 (4.6; 7.6)	11.0 (1.3)	60.0	15.4 (9.6; 21.3)	1.8 (1.4; 2.1)	3.9 (2.5; 5.3)
UEP only (*n* = 35)	3.6 (2.4; 4.8)	10.4 (1.4)	71.4	6.2 (3.2; 9.3)	1.2 (1.0; 1.4)	2.3 (1.5; 3.1)
LEP only (*n* = 290)	29.5 (26.7; 32.4)	10.6 (1.4)	45.9	18.4 (15.7; 21.0)	2.3 (2.1; 2.5)	3.5 (3.1; 4.0)
SP+UEP (*n* = 16)	1.7 (0.9; 2.5)	10.6 (1.2)	58.2	19.4 (6.3; 32.4)		
SP				15.4 (1.9; 28.9)	1.7 (1.2; 2.2)	4.0 (0.6; 7.3)
UEP				6.7 (2.3; 11.1)	1.2 (0.8; 1.4)	2.5 (0.9; 4.1)
SP+LEP (*n* = 129)	13.2 (11.1; 15.3)	10.8 (1.4)	51.5	26.9 (22.9; 31.0)		
SP				11.7 (8.7; 14.7)	1.8 (1.6; 2.0)	2.9 (2.3; 3.4)
LEP				18.3 (14.5; 22.0)	2.6 (2.3; 3.0)	3.1 (2.6; 3.5)
UEP+LEP (*n* = 94)	9.6 (7.8; 11.4)	10.8 (1.3)	53.2	24.1 (19.9; 28.3)		
UEP				6.2 (5.0; 7.5)	1.4 (1.2; 1.6)	2.0 (1.7; 2.3)
LEP				18.8 (14.7; 22.9)	2.8 (2.4; 3.2)	2.9 (2.5; 3.4)
SP+UEP+LEP (*n* = 75)	7.7 (6.0; 9.4)	11.0 (1.3)	68.4	40.2 (24.4; 46.1)		
SP				14.7 (9.7; 19.7)	2.1 (1.8; 2.5)	3.2 (2.2; 3.8)
UEP				7.2 (5.5; 8.9)	1.6 (1.4; 1.9)	1.9 (1.6; 2.2)
LEP				22.4 (17.6; 27.1)	3.4 (2.4; 3.4)	2.9 (2.4; 3.4)

*SP* spinal pain, *LEP* lower extremity pain, *UEP* Upper extremity pain, *CI* confidence interval, *SD* standard deviation

#### Proportion of weeks with pain

Overall, the children reporting pain from more sites reported pain more often than children with pain at only one site. Indeed, the total proportion of pain weeks approached the sum of weeks from individual pain sites, suggesting limited overlap of pain, i.e. in many cases the pain from different sites did not occur simultaneously (Table 2).

Generally, these patterns remained for the other two school years (Add. file 1).

#### Number and length of pain episodes

In year 1, pain from the LE occurred most often either alone or in combination with pain from other sites with 2.8 to 3.4 episodes during school year 1, depending on group. Spinal pain was responsible for the longest episodes with episode lengths from 2.9 to 4.0 weeks, depending on group. Thus, pain from the UE represented the fewest recurrences and the shortest episode duration (Table 2).

For the two following years, LE remained the most common area with pain. In year 2, spinal pain episodes were the longest in all groups except for the group with pain at all three sites where LE pain episodes were longer. For year 3, there was no consistent pattern for length of episodes (Add. file 1).

### Results for ‘At least three pain weeks’ during a school year

#### Distribution of children into eight pain groups

Using this definition, 39.1% of the children experienced pain during school year 1. The most common pain site was again the LE (29.8%), followed by spinal pain (11.6%), and UE (4.3%). One third of the children had pain in one region only, most commonly in the LEs (24.0%). Only 6.0% of the children experienced pain in more than one region, again with ´Spinal and LE pain’ as the most frequent pain combination (3.7%). The distribution of the subgroups is shown in Fig. 3b.

These patterns remained for the other two school years with 37.7 and 36.9% reporting pain during year 2 and 3, respectively, LE pain being most common, and 7% reporting pain at more than one site (Add. file 1).

#### Sex

Most pain groups were small, which makes an investigation of sex differences difficult. However, a distinct difference could be observed for spinal pain only with a higher frequency in girls (Fig. 3b).

In year 2 and 3, there were more boys than girls with LE pain only, while the girls again were most prevalent in all other groups. Differences were small and not statistically significant (Add. file 1).

#### Age

Generally, there were no differences in mean age between the sub-groups (Table 3 and Add. file 1).

**Table 3:** Description of Danish children aged 8 to 16 years during school year 1. Reported by pain group. Pain was defined as **at least three pain weeks** from a body region during a school year

Pain site(s)	Proportion % (95%CI)	Age Years (SD)	Sex % female	Weeks with pain % (95%CI)	Number of episodes (95%CI)	Length of episodes Weeks (95%CI)
No pain (*n* = 598)	60.9 (57.0; 63.9)	10.6 (1.4)	48.5	0	0	-
SP only (*n* = 69)	7.0 (5.6; 8.8)	11.1 (1.4)	72.5	25.0 (19.3; 30.8)	2.4 (2.1; 2.8)	4.5 (3.4; 5.6)
UEP only (*n* = 20)	2.0 (1.3; 3.1)	11.1 (1.3)	60.0	18.7 (14.5; 22.8)	2.6 (1.8; 3.4)	3.2 (2.4; 4.0)
LEP only (*n* = 236)	24.0 (21.5; 26.8)	10.8 (1.3)	52.5	33.8 (30.7; 36.8)	3.6 (3.3; 3.9)	4.1 (3.8; 4.5)
SP+UEP (*n* = 2)	0.2 (0.1; 0.8)	10.5 (0.7)	0	58.0 (-)		
SP				54.5 (-)	2 (-)	12 (-)
UEP				22.7 (-)	2 (-)	5 (-)
SP+LEP (*n* = 36)	3.7 (2.7; 5.0)	11.1 (1.2)	55.6	51.3 (43.5; 59.1)		
SP				31.9 (23.4; 40.3)	3.3 (2.6; 3.9)	4.3 (3.6; 4.8)
LEP				30.4 (23.0; 37.9)	4.1 (3.6; 4.8)	3.3 (2.5; 4.1)
UEP+LEP (*n* = 14)	1.4 (0.8; 2.4)	10.5 (1.3)	78.6	48.1 (37.1; 59.1)		
UEP				16.6 (12.1; 21.0)	1.9 (1.4; 2.4)	3.9 (2.7; 5.2)
LEP				35.6 (23.7; 47.6)	4.0 (2.9; 5.1)	3.9 (2.6; 5.2)
SP+UEP+LEP (*n* = 7)	0.7 (0.3; 1.5)	11.1 (1.2)	71.4	75.0 (60.8; 89.1)		
SP				38.0 (9.3; 66.7)	4.3 (2.7; 5.9)	3.9 (2.1; 5.7)
UEP				19.5 (10.5; 28.6)	3.0 (1.4; 4.6)	2.9 (2.1; 3.6)
LEP				30.8 (9.3; 52.2)	3.7 (2.7; 4.7)	3.6 (1.1; 6.2)

*SP* spinal pain, *LEP* lower extremity pain, *UEP* Upper extremity pain, *CI* confidence interval, *SD* standard deviation

#### Proportion of weeks with pain

Again, the children with pain from more sites reported pain about twice as often compared to children with pain at only one site. (Table 3).

No additional information could be derived from the other two school years (Add. file 1).

#### Number and length of pain episodes

Most children reported pain from the LE with 3.6 to 4.1 episodes during school year 1, depending on group. Spinal pain was responsible for the longest episodes with episode lengths from 3.9 to 4.5 weeks, depending on group. Thus again, pain from the UE represented the fewest recurrences and the shortest episode duration (Table 3).

For the two following years, LE pain was also the most frequent, but there was no consistent pattern for length of episodes (Add. file 1).

### Missing SMS responses

Overall, our data imputation schemes did not change the results of any of the analyses (Additional file 2).

## Discussion

### Summary of findings

In a 3-year cohort study of Danish school children aged 8 to 14 years at baseline, we found that children with pain most frequently reported pain in one region, most frequently in the LE, however, many children reported pain in more than one region during a school year. When pain was defined as ‘at least one pain week in each region during a school year’, 30% reported pain in more than one region versus only 6% when pain was defined as ‘at least three pain weeks in each region during a school year’ indicating that most episodes were of short duration. About 70% reported at least 1 week with pain during a school year and 30% reported at least 3 weeks. Most children with spinal pain or with pain in more than one region were girls.

### Comparison to previous literature

We found that during a school year around 30% of the children reported at least one pain week in more than one region, which is higher when compared to two other school-based cohort studies. Holden et al. found that about 20% had ‘Multisite bodily pain’ [10] based on self-reported data for point prevalence, which is expected to be lower than in the current study, based on 1 year prevalence. This might have been partly counterbalanced by data in our study being parentally reported, since parents appear to report lower prevalence rates of pain than the children themselves [29].

Approximately 6% of the children reported pain in more than one region lasting at least 3 weeks during a school year, which is similar to findings in two other studies [30, 31]. Hoftun et al. found MSK pain in at least three anatomical pain sites in 8.5% of adolescents aged 13 to 19 years [30] and Mikkelsson et al. found the prevalence of widespread pain that was not exclusively musculoskeletal pain increasing from 7% in children aged 10–12 years, 9% when children were 11–13 years, to 15% in children aged 14 to 16 years [31]. In both studies somewhat different duration and frequency of pain were used, however, despite our lenient definition of pain, the affected children reported pain in about 50% of the weeks or more during a school year and thus the pain definitions may be comparable.

We found that the majority of children with pain in more than one region were girls, which is similar to findings from other studies [4, 10, 30–32].

### Strengths and weaknesses

In this study, parents reported on behalf of the children, which may bias results. However participating children, when asked, more often reported pain that was not reported by their parents, whereas the opposite was rarely the case, according to a validity study nested within the CHAMPS Study-DK [33]. Thus, our estimates are probably conservative when compared to studies relying on child-reported data. Concordance was better for pain of greater intensity, indicating that parents did not report minor pain, which was also reported by Sundblad et al. [29], whereas better concordance was found when children were more severely ill [34]. Potentially, this is a limitation in this study, if parents also were reluctant to report secondary pain of short duration.

When pain was defined as at least 1 week in each region during a school year, around 30% reported pain in more than one region, versus 6% if at least 3 pain weeks in each region was needed. Thus, it may be argued that the difference in proportion between these two groups would be smaller if parents were reluctant to report secondary pain of short duration.

Data collection via mobile phone text messages is known to be practical and user-friendly [35, 36] with responses comparable to telephone interviews [35]. An expected limitation was that parents answered SMS questions continuously every week for more than 5 years, and therefore some response fatigue could have occurred. However, this did not seem to be the case since the response rate was high and did not decrease during the study period.

Major strengths of this study include the large prospective population-based cohort, the high response rate of the text messages, and the short recall period.

### Implications

Many children report pain in more than one body region during a school year. Whether the different pain regions are to be regarded as individual entities or part of a more general MSK syndrome, is still unknown. Some degree of overlap is to be expected by chance, and it is known from studies of both adolescent and adult populations, that disorders tend to cluster in some individuals [37, 38]. In adolescents it has been found that pain in one part of the spine increased the risk of experiencing pain in other parts within the following 2 years, so it is also possible that MSK pain in one region increases the risk of pain in other regions [39].. This could have clinical implications, as knowledge of such a sequence will increase the incentive to implement treatment at the initial stage of the potential cascade. Therefore, an interesting next step could be to map the sequences of pain appearance to potentially identify the optimal time and body region with regard to prevention and early effective treatment.

## Conclusion

Danish schoolchildren often experienced pain at more than one pain site during a schoolyear, and a significantly larger proportion of girls than boys reported pain in all three regions. This could indicate that, at least in some instances, the musculoskeletal system should be regarded as one entity, both for clinical and research purposes.

## Supplementary information

**Additional file 1.** Illustrations with information about school years 2 and 3.

**Additional file 2. **Sensitivity analyses of missing data. Primary data: pain defined as at least one pain week in each site during school year 1. *n*=982, of which 280 children did not report pain. Sensitivity analyses of missing data. Primary data: pain were defined as at least three pain weeks within school year 1. *n*=982, of which 598 children did not report pain.

## Data Availability

Data are available only upon request from the CHAMPS Study-DK Steering Committee due to legal and ethical restrictions. Interested parties may contact Dr. Niels Wedderkopp (nwedderkopp@health.sdu.dk) and the following information will be required at the time of application: a description of how the data will be used, securely managed, and permanently deleted.

## Abbreviations

CHAMPS Study-DK: Childhood Health, Activity and Motor Performance School Study
CI: Confidence interval
LE: Lower extremity
MSK: Musculoskeletal
SMS: Mobile phone text message
UE: Upper extremity

## Content of publication

Written informed consent was obtained from the participants.
