# Supplementary material for: Musculoskeletal pain distribution in 1,000 Danish schoolchildren aged 8–16 years
Source: Chiropr Man Therap. 2020 Aug 4;28:45. doi: 10.1186/s12998-020-00330-9 (PMC7401207; doi:10.1186/s12998-020-00330-9)
Supplement: Supplementary file 1 — Additional file 1. Illustrations with information about school years 2 and 3. [file 12998_2020_330_MOESM1_ESM.docx]

**Additional file 1 Illustrations with information about school years 2 and 3.**

Proportion of Danish children aged 8 to 16 years with either a) at least one week with pain or b) at least three weeks with pain in each anatomical region during *school year 2*. Presented by sex and with 95% confidence interval (CI). UE: upper extremity, LE: lower extremity

Proportion of Danish children aged 8 to 16 years with either a) at least one week with pain or b) at least three weeks with pain in each anatomical region during *school year 3*. Presented by sex and with 95% confidence interval (CI). UE: upper extremity, LE: lower extremity

Description of Danish children aged 8 to 16 years regarding mean age, proportion of weeks with pain, mean number and mean length of episodes during **school year 2.** Reported by pain group.

Pain was defined as **at least one pain week** from a body region during a school year.

| Pain site(s) | Proportion  % (95%CI) | Age  Years (SD) | Sex  % female | Weeks with pain  % (95%CI) | Number of  episodes (95%CI) | Length of  episodes  Weeks (95%CI) |
| --- | --- | --- | --- | --- | --- | --- |
| No pain (n=298) | 27.1 (24.5; 29.8) | 11.4 (1.5) | 56.0 | 0 | 0 | - |
| SP only (n=96) | 8.7 (7.2; 7.6) | 11.9 (1.5) | 56.3 | 16.0 (11.2; 20.8) | 1.7 (1.5; 2.0) | 4.3 (3.2; 5.5) |
| UEP only (n=41) | 3.7 (2.8; 5.0) | 11.7 (1.5) | 58.3 | 6.4 (3.2; 9.6) | 1.3 (1.1; 1.5) | 2.3 (1.4; 3.2) |
| LEP only (n=294) | 26.7 (24.2; 29.4) | 11.5 (1.3) | 42.9 | 18.4 (15.6; 21.1) | 2.0 (1.9; 2.2) | 4.2 (3.6; 4.7) |
| SP+UEP  (n=24)  SP  UEP | 2.2 (1.5; 3.2) | 11.5 (1.4) | 58.3 | 23.6 (19.4; 36.7)  19.5 (6.0; 32.9)  6.2 (3.0; 9.3) | 1.8 (1.2; 2.7)  1.3 (1.0; 1.7) | 5.1 (2.5; 7.7)  2.1 (1.3; 3.0) |
| SP+LEP  (n=150)  SP  LEP | 13.6 (11.7; 15.8) | 11.8 (1.5) | 49.3 | 30.4 (26.2; 34.6)  15.3 (11.9; 18.6)  18.5 (15.0; 22.0) | 1.9 (1.6; 2.2)  2.5 (2.2; 2.8) | 3.7 (2.2; 2.8)  3.4 (2.8; 3.9) |
| UEP+LEP  (n=97)  UEP  LEP | 8.0 (7.3; 10.6) | 11.4 (1.2) | 55.7 | 22.6 (18.8; 26.4)  6.9 (5.1; 8.7)  16.3 (12.8; 19.8) | 1.5 (1.3; 1.7)  2.8 (2.4; 3.2) | 2.1 (1.7; 2.5)  2.7 (2.3; 3.1) |
| SP+UEP+LEP  (n=100)  SP  UEP  LEP | 9.1 (7.5; 10.9) | 11.6 (1.4) | 63.0 | 35.7 (30.8; 40.5)  11.8 (8.6; 14.9)  9.0 (6.8; 11.1)  20.1 (15.5; 24.7) | 2.2 (1.8; 2.6)  1.8 (1.6; 2.1)  2.9 (2.5; 3.2) | 2.5 (1.9; 3.0)  2.2 (1.8; 2.6)  3.2 (2.6; 3.9) |

SP: spinal pain, LEP: lower extremity pain, UEP: Upper extremity pain, CI: confidence interval, SD: standard deviation

Description of Danish children aged 8 to 16 years regarding mean age, proportion of weeks with pain, mean number and mean length of episodes during **school year 2.** Reported by pain group.

Pain was defined as **at least three pain weeks** from a body region during a school year.

| Pain site(s) | Proportion  % (95%CI) | Age  Years (SD) | Sex  % female | Weeks with pain  % (95%CI) | Number of  episodes (95%CI) | Length of  episodes  Weeks (95%CI) |
| --- | --- | --- | --- | --- | --- | --- |
| No pain (n=585) | 62.3 (59.4; 65.1) | 11.5 (1.4) | 50.2 | 0 | 0 | - |
| SP only (n=91) | 8.3 (6.8; 10.1) | 12.0 (1.3) | 58.2 | 30.6 (24.9; 35.7) | 2.6 (2.2; 2.9) | 5.4 (4.4; 6.5) |
| UEP only (n=24) | 2.2 (1.5; 3.2) | 12.0 (1.3) | 58.3 | 24.8 (17.7; 32.0) | 2.9 (2.4; 3.3) | 4.0 (2.9; 5.0) |
| LEP only (n=221) | 20.1 (17.8; 22.6) | 11.6 (1.3) | 49.8 | 34.2 (30.9; 37.4) | 3.3 (3.1; 3.6) | 4.7 (4.2; 5.3) |
| SP+UEP (n=6)  SP  UEP | 0.5 (0.2; 1.2) | 10.8 (1.8) | 83.3 | 57.2 (26.6; 87.9)  41.7 (0.7; 82.6)  25.7 (19.0; 32.4) | 3.2 (1.5; 4.8)  3.3 (1.8; 4.9) | 6.1 (1.5; 10.6)  3.6 (1.8; 5.3) |
| SP+LEP (n=54)  SP  LEP | 4.9 (3.8; 6.4) | 11.8 (1.4) | 70.4 | 54.9 (48.5; 61.3)  28.7 (22.8; 34.6)  35.6 (28.9; 32.3) | 3.2 (2.5; 3.9)  3.7 (3.2; 4.3) | 4.2 (3.2; 5.1)  4.4 (3.5; 5.3) |
| UEP+LEP (n=11)  UEP  LEP | 1.0 (0.6; 1.8) | 11.4 (1.2) | 72.7 | 53.6 (39.2; 67.9)  20.4 (12.8; 28.0)  36.0 (18.0; 53.9) | 2.9 (2.0; 3.8)  3.6 (2.4; 4.9) | 3.2 (2.2; 4.3)  4.9 (2.1; 7.0) |
| SP+UEP+LEP  (n=8)  SP  UEP  LEP | 0.7 (0.4; 1.4) | 12.0 (1.2) | 50.0 | 59.5 (39.4; 79.7)  29.6 (2.3; 57.0)  20.9 (12.4; 29.4)  36.7 (11.7; 61.7) | 3.4 (1.4; 5.3)  2.9 (0.9; 4.8)  3.5 (2.2; 4.8) | 4.0 (0.5; 7.5)  3.3 (1.8; 4.9)  4.8 (2.6; 7.1) |

SP: spinal pain, LEP: lower extremity pain, UEP: Upper extremity pain, CI: confidence interval, SD: standard deviation

Description of Danish children aged 8 to 16 years regarding mean age, proportion of weeks with pain, mean number and mean length of episodes during **school year 3**. Reported by pain group.

Pain was defined as **at least one pain week** from a body region during a school year.

| Pain site(s) | Proportion  % (95%CI) | Age  Years (SD) | Sex  % female | Weeks with pain  % (95%CI) | Number of  episodes (95%CI) | Length of  episodes  Weeks (95%CI) |
| --- | --- | --- | --- | --- | --- | --- |
| No pain (n=318) | 30.8 (28.0; 33.7) | 12.6 (1.5) | 49.7 | 0 | 0 | - |
| SP only (n=100) | 9.7 (8.0; 11.6) | 12.9 (1.3) | 53.0 | 16.6 (11.7; 21.5) | 1.7 (1.5; 1.9) | 4.5 (3.3; 5.8) |
| UEP only (n=44) | 4.3 (3.2; 5.7) | 13.0 (1.3) | 54.6 | 10.4 (5.6; 15.1) | 1.3 (1.1; 1.5) | 3.6 (1.9; 5.3) |
| LEP only (n=262) | 25.4 (22.8; 28.1) | 12.4 (1.4) | 52.3 | 19.2 (16.3; 22.1) | 2.0 (1.8; 2.2) | 4.5 (3.8; 5.3) |
| SP+UEP  (n=22)  SP  UEP | 2.1 (1.4; 3.2) | 12.3 (1.6) | 59.1 | 17.7 (11.4; 24.0)  9.0 (4.9; 13.1)  9.3 (4.0; 14.6) | 1.9 (1.2; 2.6)  1.4 (1.0; 1.8) | 2.2 (1.3; 3.0)  3.1 (1.6; 4.7) |
| SP+LEP  (n=126)  SP  LEP | 12.2 (10.3; 14.3) | 12.5 (1.4) | 46.0 | 33.4 (28.2; 38.6)  14.5 (11.0; 17.9)  21.6 (17.1; 26.2) | 2.0 (1.7; 2.3)  2.4 (2.1; 2.7) | 3.3 (2.7; 3.9)  4.2 (3.3; 5.0) |
| UEP+LEP  (n=87)  UEP  LEP | 8.4 (6.9; 10.3) | 12.2 (1.3) | 55.2 | 21.3 (17.5; 25.1)  9.5 (6.9; 12.0)  12.4 (9.5; 15.2) | 1.5 (1.3; 1.7)  2.1 (1.8; 2.4) | 3.0 (2.3; 3.7)  2.8 (2.2; 3.3) |
| SP+UEP+LEP  (n=74)  SP  UEP  LEP | 7.2 (5.7; 8.9) | 12.5 (1.3) | 64.9 | 39.0 (30.8; 40.5)  18.5 (13.2; 23.8)  9.2 (6.2; 12.7)  20.7 (15.0; 26.3) | 2.3 (1.9; 2.7)  1.7 (1.4; 2.1)  2.9 (2.5; 3.4) | 3.7 (2.6; 4.7)  2.5 (1.9; 3.1)  3.2 (2.6; 3.8) |

SP: spinal pain, LEP: lower extremity pain, UEP: Upper extremity pain, CI: confidence interval, SD: standard deviation

Description of Danish children aged 8 to 16 years regarding mean age, proportion of weeks with pain, mean number and mean length of episodes during **school year 3**. Reported by pain group.

Pain was defined as **at least three pain weeks** from a body region during a school year.

| Pain site(s) | Proportion  % (95%CI) | Age  Years (SD) | Sex  % female | Weeks with pain  % (95%CI) | Number of  episodes (95%CI) | Length of  episodes  Weeks (95%CI) |
| --- | --- | --- | --- | --- | --- | --- |
| No pain (n=652) | 63.1 (60.1; 66.0) | 12.5 (1.4) | 48.6 | 0 | 0 | - |
| SP only (n=82) | 7.9 (6.4; 9.8) | 12.9 (1.3) | 56.1 | 29.0 (23.3; 34.9) | 2.6 (2.3; 2.9) | 5.2 (4.0; 6.3) |
| UEP only (n=29) | 2.8 (2.0; 4.0) | 13.0 (1.4) | 48.3 | 27.4 (20.7; 43.0) | 2.3 (1.8; 2.8) | 5.5 (3.9; 7.2) |
| LEP only (n=196) | 19.0 (16.7; 21.5) | 12.4 (1.4) | 57.1 | 33.9 (30.3; 37.6) | 3.0 (2.7; 3.2) | 5.2 (4.6; 5.9) |
| SP+UEP (n=5)  SP  UEP | 0.5 (0.2; 1.2) | 12.0 (2.0) | 100.0 | 51.3 (28.2; 74.4)  32.2 (3.5; 60.9)  21.7 (11.1; 32.4) | 3.0 (2.1; 3.9)  3.6 (0; 7.3) | 4.9 (1.8; 8.0)  2.8 (1.7; 3.9) |
| SP+LEP (n=49)  SP  LEP | 4.7 (3.6; 6.2) | 12.7 (1.4) | 59.2 | 56.8 (49.1; 64.1)  33.0 (26.4; 39.6)  34.7 (27.1; 41.7) | 3.5 (2.9; 4.1)  3.6 (3.0; 4.2) | 4.4 (3.4; 5.3)  4.4 (3.4; 5.4) |
| UEP+LEP (n=12)  UEP  LEP | 1.2 (0.7; 2.0) | 12.3 (1.5) | 75.0 | 50.9 (37.6; 64.2)  25.0 (15.7; 34.3)  30.1 (19.1; 41.1) | 2.3 (1.5; 3.2)  3.0 (1.7; 4.3) | 4.9 (2.5; 7.4)  4.6 (2.8; 6.4) |
| SP+UEP+LEP  (n=8)  SP  UEP  LEP | 0.8 (0.4; 1.5) | 12.3 (1.6) | 87.5 | 76.9 (62.8; 91.0)  34.6 (10.4; 58.9)  33.2 (9.3; 57.2)  51.1 (22.1; 80.0) | 2.9 (1.7; 4.1)  2.8 (1.3; 4.2)  3.6 (2.6; 8.6) | 5.5 (2.1; 9.9)  5.5 (2.6; 8.6)  6.4 (3.4; 9.4) |

SP: spinal pain, LEP: lower extremity pain, UEP: Upper extremity pain, CI: confidence interval, SD: standard deviation
