# Supplementary material for: Musculoskeletal pain distribution in 1,000 Danish schoolchildren aged 8–16 years
Source: Chiropr Man Therap. 2020 Aug 4;28:45. doi: 10.1186/s12998-020-00330-9 (PMC7401207; doi:10.1186/s12998-020-00330-9)
Supplement: Supplementary file 2 — Additional file 2. Sensitivity analyses of missing data. Primary data: pain defined as at least one pain week in each site during school year 1. n=982, of which 280 children did not report pain. Sensitivity analyses of missing data. Primary data: pain were defined as at least three pain weeks within school year 1. n=982, of which 598 children did not report pain. [file 12998_2020_330_MOESM2_ESM.docx]

**Additional file 2**

**Imputation of missing mobile phone text message responses (SMS responses)**

1. In the analysis described below as ‘primary data’, the missing SMS answers were imputed according to the defined decision rules.

2. In the analysis described below as ‘missing imputed as the same as the last SMS answer (+pain)’, all missing SMS answers were imputed as the same as the last available SMS answer.

3. In the analysis described below as ‘missing imputed as ‘4’ (no pain)’, all missing SMS answers were imputed as ‘no pain’.

A sensitivity analysis was performed to estimate the impact of the decision rules. For scenario two and three, mean and median number of episodes, and mean and median length of episodes were calculated, and results were compared with the primary data.

Analyses were performed for school year 1, for both pain definitions.

| **Additional file 2** Sensitivity analyses of missing data. Primary data: pain defined as at least one pain week in each site during school year 1. n=982, of which 280 children did not report pain. | | | | | | | | | | | | | |
| --- | --- | --- | --- | --- | --- | --- | --- | --- | --- | --- | --- | --- | --- |
|  | Primary data | | | | Missing imputed as the same as last answer (+pain) | | | | Missing imputed as ’4’ (no pain) | | | | |
|  | Number of episodes | | Length of episodes in weeks | | Number of episodes | | Length of episodes in weeks | | Number of episodes | | | Length of episodes in weeks | |
|  | Mean  (95% CI) | Median  (25%-75%) | Mean  (95% CI) | Median (25%-75%) | Mean  (95% CI) | Median (25%-75%) | Mean  (95% CI) | Median  (25%-75%) | Mean  (95% CI) | | Median (25%-75%) | Mean  (95% CI) | Median (25%-75%) |
| **Spine** (n=60) | | | | |  |  |  |  |  | |  |  |  |
|  | 1.8  (1.4-2.1) | 1 (1-2) | 3.9  (2.5-5.3) | 1 (1-3) | 1.96  (1.67-2.25) | 1 (1-2) | 3.92  (2.50-5.34) | 1 (1-3) | 2.09  (1.82-2.37) | | 1 (1-3) | 3.33  (2.55-4.11) | 1.5 (1-4) |
| **Upper extremity** (n=35) | | | | |  | | | | |  | | | |
|  | 1.2  (1.0-1.4) | 1  (1-1) | 2.3  (1.5-3.1) | 1  (1-3) | 1.19  (1.05-1.33) | 1  (1-1) | 2.33  (1.55-3.12) | 1  (1-3) | 1.27  (1.09-1.45) | | 1  (1-1) | 1.09  (0.96-1.22) | 1  (1-1) |
| **Lower extremity** (n=290) | | | | | | | | | | | | | |
|  | 2.3  (2.1-2.5) | 2  (1-3) | 3.5  (3.1-4.0) | 1  (1-4) | 2.26  (2.13-2.38) | 2  (1-3) | 3.62  (3.20-4.04) | 2  (1-4) | 2.36  (2.23-2.49) | | 2  (1-3) | 3.32  (3.01-3.63) | 1  (1-4) |
| **Spinal and UE pain** (n=17) | | | | |  |  |  |  |  | |  |  |  |
| Spine | 1.7  (1.2-2.2) | 1 (1-2) | 4.0  (0.6-7.3) | 1 (1-2) | 1.66  (1.30-2.01) | 1 (1-2) | 4.0  (0.68-7.32) | 1 (1-2) | 1.8  (0.76-2.84) | | 1 (1-2) | 3.65  (1.36-5.93) | 1 (1-2) |
| UE | 1.2  (0.8-1.4) | 1 (1-1) | 2.5  (0.9-4.1) | 1 (1-2.5) | 1.15  (0.98-1.32) | 1 (1-1) | 2.55  (0.92-4.18) | 1 (1-2.5) | 1.15  (0.98-1.32) | | 1 (1-2) | **1**  (-) | 1 (1-1) |
| **Spinal and LE pain** (n=130) | | | | |  |  |  |  |  | |  |  |  |
| Spine | 1.8  (1.6-2.0) | 1 (1-2) | 2.9  (2.3-3.4) | 1 (1-3) | 1.90  (1.73-2.07) | 1 (1-2) | 2.89  (2.29-3.49) | 1 (1-3) | 2.01  (1.83-2.20) | | 1 (1-3) | 2.69  (2.26-3.12) | 1 (1-3) |
| LE | 2.6  (2.3-3.0) | 2 (1-3) | 3.1  (2.6-3.5) | 1 (1-3) | 2.56  (2.36-2.76) | 2 (1-3) | 3.13  (2.68-3.59) | 2 (1-3) | 2.65  (2.45-2.85) | | 2 (1-4) | 2.85  (2.50-3.20) | 1 (1-3) |
| **UE and LE pain** (n=94) | | | | |  |  |  |  |  | |  |  |  |
| UE | 1.4  (1.2-1.6) | 1 (1-1) | 2.0  (1.7-2.3) | 1 (1-2) | 1.46  (1.29-1.63) | 1 (1-2) | 2.03  (1.70-2.36) | 1 (1-2) | 1.48  (1.31-1.65) | | 1 (1-2) | **1.05**  (1.00-1.11) | 1(1-1) |
| LE | 2.8  (2.4-3.2) | 2 (1-4) | 2.9  (2.5-3.4) | 1 (1-3) | 2.69  (2.46-2.93) | 2 (1-4) | 2.99  (2.51-3.47) | 2 (1-3) | 2.77  (2.53-3.00) | | 2 (1-4) | 2.81  (2.42-3.20) | 2 (1-3) |
| **All pain sites** (n=94) | | | | |  |  |  |  |  | |  |  |  |
| Spine | 2.1  (1.8-2.5) | 1 (1-3) | 3.2  (2.2-3.8) | 1 (1-3) | 2.12  (1.90-2.35) | 2 (1-3) | 3.04  (2.23-3.84) | 1 (1-3) | 2.22  (1.99-2.45) | | 2 (1-3) | 2.83  (2.26-3.41) | 1 (1-3) |
| UE | 1.6  (1.4-1.9) | 1 (1-2) | 1.9  (1.6-2.2) | 1 (1-2) | 1.72  (1.51-1.93) | 1 (1-2) | 2.02  (1.73-2.32) | 1 (1-2) | 1.87  (1.60-2.13) | | 1 (1-2) | **1.05**  (1.01-1.08) | 1 (1-1) |
| LE | 3.4  (2.9-3.9) | 3 (2-4) | 2.9  (2.4-3.4) | 1 (1-3) | **2.84**  (2.60-3.07) | 2 (1-4) | 2.92  (2.45-3.40) | 2 (1-3) | 2.93  (2.70-3.16) | | 2 (1-4) | 2.70  (2.32-3.07) | 1 (1-3) |
| bold: result different compared with results in the primary data  CI: confidence interval | | | | | | | | | | | | | |

| **Additional file 2** Sensitivity analyses of missing data. Primary data: pain were defined as at least three pain weeks within school year 1. n=982, of which 598 children did not report pain. | | | | | | | | | | | | | | |
| --- | --- | --- | --- | --- | --- | --- | --- | --- | --- | --- | --- | --- | --- | --- |
|  | Primary data | | | | | Missing imputed as the same as last answer (+pain) | | | | Missing imputed as ’4’ (no pain) | | | | |
|  | Number of episodes | | Length of episodes in weeks | | | Number of episodes | | Length of episodes in weeks | | Number of episodes | | | Length of episodes in weeks | |
|  | Mean  (95% CI) | Median  (25%-75%) | Mean  (95% CI) | Median (25%-75%) | | Mean  (95% CI) | Median (25%-75%) | Mean  (95% CI) | Median  (25%-75%) | Mean  (95% CI) | | Median (25%-75%) | Mean  (95% CI) | Median (25%-75%) |
| **Spine** (n=69) | | | | | |  |  |  |  |  | |  |  |  |
|  | 2.4  (2.1-2.8) | 2 (1-3) | 4.5  (3.4-5.6) | 2 (1-5) | | 2.20  (1.99-2.41) | 2 (1-3) | 4.57  (3.47-5.66) | 2 (1-5) | 2.31  (2.11-2.51) | | 2 (1-3) | 3.95  (3.29-4.60) | 2 (1-5) |
| **Upper extremity** (n=20) | | | | |  | | | | | |  | | | |
|  | 2.6  (1.8-3.4) | 1.5  (2-3) | 3.2  (2.4-4.0) | 2  (1-4) | | 2.37  (1.91-2.82) | 2  (1-3) | 3.19  (2.38-4.00) | 2  (1-4) | 2.38  (1.95-2.81) | | 2  (1-3) | 1.11  (1.01-1.21) | 1  (1-1) |
| **Lower extremity** (n=236) | | | | | | | | | | | | | | |
|  | 3.6  (3.3-3.9) | 2  (2-5) | 4.1  (3.8-4.5) | 2  (1-5) | | **2.92**  (2.79-3.06) | 2  (1-4) | 4.22  (3.85-4.60) | 2  (1-5) | **3.01**  (2.88-3.14) | | 3  (1-4) | 3.82  (3.54-4.10) | 2  (1-5) |
| **Spinal and UE pain** (n=2) | | | | | |  |  |  |  |  | |  |  |  |
| Spine | 2  (-) | 2 (1-3) | 12.0  (-) | 1.5 (1-23) | | 1.75  (0.23-3.27) | 1.5 (1-2.5) | 12  (-) | 1.5 (1-23) | 1.8  (0.76-2.84) | | 2 (1-2) | 9.4  (-4.88-23.68) | 2 (1-17) |
| UE | 2  (-) | 2 (1-3) | 5  (-5.6-15.6) | 2 (1.5-8.5) | | 1.75  (0.23-3.27) | 1.5 (1-2.5) | 5  (-5.63-15.63) | 2 (1.5-8.5) | 1.75  (0.23-3.27) | | 1.5 (1-2.5) | 1  (-) | 1 (1-1) |
| **Spinal and LE pain** (n=36) | | | | | |  |  |  |  |  | |  |  |  |
| Spine | 3.25  (2.6-3.9) | 3 (2-5) | 4.3  (3.1-5.5) | 3 (1-5) | | 2.70  (2.39-3.01) | 2 (1-4) | 4.33  (3.14-5.53) | 3 (1-5) | 2.90  (2.57-3.22) | | 2 (1-4) | 3.90  (3.12-4.67) | 3 (1-5) |
| LE | 4.1  (3.6-4.8) | 4 (2.5-5) | 3.3  (2.5-4.1) | 2 (1-3) | | **3.09**  (2.76-3.42) | 3 (2-4) | 3.35  (2.55-4.16) | 2 (1-3) | **3.2**  (2.87-3.53) | | 3 (2-4) | 3.05  (2.45-3.64) | 2 (1-3) |
| **UE and LE pain** (n=14) | | | | | |  |  |  |  |  | |  |  |  |
| UE | 1.86  (1.4-2.4) | 2 (1-2) | 3.9  (2.7-5.2) | 4 (1-5) | | 1.62  (1.29-1.94) | 1 (1-2) | 3.96  (2.69-5.23) | 4 (1-5) | 1.67  (1.34-2.00) | | 1 (1-2) | 1  (-) | 1 (1-1) |
| LE | 4.0  (2.9-5.1) | 3 (3-6) | 3.9  (2.6-5.2) | 2 (1-4) | | **2.91**  (2.45-3.37) | 3 (1.5-4) | 3.91  (2.60-5.22) | 2 (1-4) | 3.15  (2.67-3.62) | | 3 (2-4) | 3.44  (2.54-4.33) | 2 (1-4) |
| **All pain sites** (n=7) | | | | | |  |  |  |  |  | |  |  |  |
| Spine | 4.3  (2.7-5.9) | 4 (4-6) | 3.9  (2.1-5.7) | 2 (1-4) | | 2.93  (2.35-3.51) | 3 (2-4) | 3.87  (2.08-5.66) | 2 (1-4) | 3  (2.42-3.58) | | 3 (2-4) | 3.68  (2.07-5.28) | 2 (1-4) |
| UE | 3  (1.4-4.6) | 2 (2-4) | 2.9  (2.1-3.6) | 2 (2-4) | | 2.43  (1.76-3.10) | 2 (1-3) | 2.95  (2.16-3.75) | 2 (2-4) | 3.13  (2.13-4.12) | | 2 (1-4) | 1.04  (0.96-1.13) | 1 (1-1) |
| LE | 3.7  (2.7-4.7) | 3 (3-4) | 3.6  (1.1-6.2) | 1.5 (1-3) | | **2.5**  (1.96-3.04) | 2 (1-3) | 3.65  (1.10-6.21) | 2 (1-3) | 2.69  (2.16-3.22) | | 3 (2-4) | 3.14  (1.65-4.62) | 2 (1-3) |
| bold: result different compared with results in the primary data  CI: confidence interval | | | | | | | | | | | | | | |
